# Fate of iatrogenic atrial septal defects following mitral transcatheter edge-to-edge repair – a subanalysis of the MITHRAS trial

**DOI:** 10.1007/s10554-022-02750-5

**Published:** 2022-11-13

**Authors:** Stephan Blazek, Matthias Unterhuber, Karl-Philipp Rommel, Karl-Patrik Kresoja, Tobias Kister, Christian Besler, Karl Fengler, Sebastian Rosch, Ingo Daehnert, Holger Thiele, Philipp Lurz, Maximilian von Roeder

**Affiliations:** 1 Department of Internal Medicine/Cardiology, Heart Center Leipzig at Leipzig University, Struempellstrasse 39, 04289 Leipzig, Germany; 2 Leipzig Heart Institute, Leipzig, Germany; 3 Department of Pediatric Cardiology, Heart Center Leipzig at Leipzig University, Leipzig, Germany

**Keywords:** Transcatheter mitral valve edge-to-edge repair, Atrial septal defect, Closure, Shunt, Heart failure

## Abstract

Persisting iatrogenic atrial septal defects (iASD) after transcatheter mitral edge-to-edge repair (M-TEER) are associated with impaired outcomes. We investigated the natural history of relevant iASDs with left-to-right shunting post-M-TEER, predictors of spontaneous closure of iASD between 1 and 6 months post-M-TEER, and outcomes (heart failure [HF] hospitalization) in patients with spontaneous closure versus those with persistent iASD 6 months post-M-TEER. Patients with a relevant iASD 1-month post-M-TEER, who were treated conservatively in the randomized controlled MITHRAS trial, underwent clinical follow-up including transesophageal echocardiography 6 months post-M-TEER. Overall, 36 patients (median 77 [interquartile range 65–81] years; 36% women) completed the 6-months follow-up. Six (17%) patients had a spontaneous closure of the iASD. The eccentricity index of the iASD 1-month after M-TEER was the strongest predictor for spontaneous closure (Odds ratio 3.78; 95% confidence interval 1.26–11.33, p = 0.01) and an eccentricity index of < 1.9 provided a sensitivity of 77% at a specificity of 83% for iASD persistence (Area under the curve 0.83, p < 0.001) within 6-months post M-TEER.

At follow-up, a numerical difference in the endpoint of HF hospitalization between the spontaneous closure and the residual shunt group (0% vs. 20%, p = 0.25) was observed. The eccentricity of the iASD was the strongest predictor for spontaneous closure at 1-months and an eccentricity index of < 1.9 is associated with a high persistence rate for 6 month after M-TEER.

* Clinical Trial Registration* ClinicalTrials.gov https://clinicaltrials.gov/ct2/show/NCT03024268 Identifier: NCT03024268.

**Graphical Abstract:**

a (red) is reflecting the mayor lengthwise dimension and b (blue) the mayor oblique dimension. The eccentricity index is calculated by dividing a through b. (Open circle) is depicting an example for a round iASD and (Open rhombus) an example for an eccentric iASD 1 month after M-TEER.
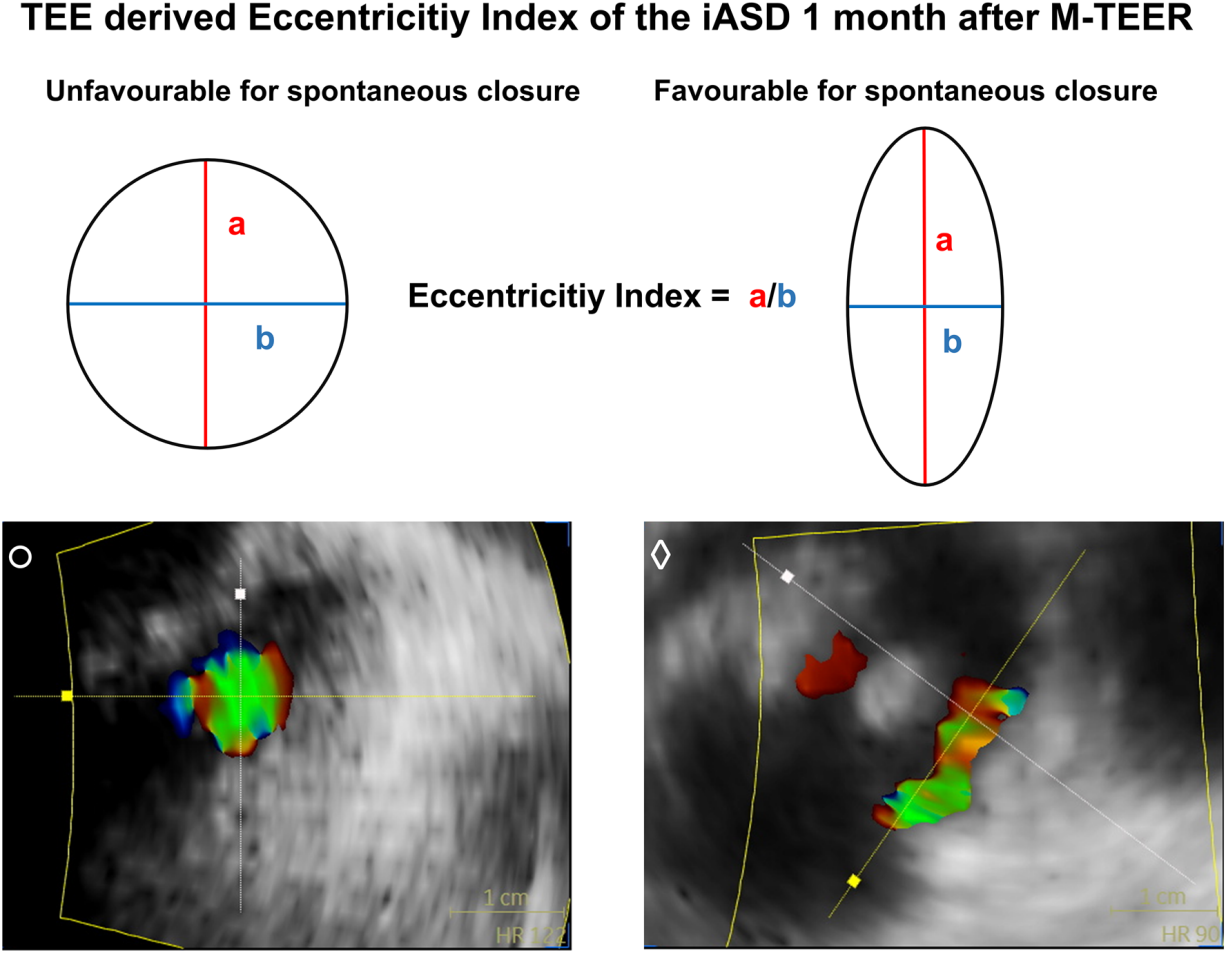

**Supplementary Information:**

The online version contains supplementary material available at 10.1007/s10554-022-02750-5.

## Introduction

Transcatheter mitral edge-to-edge repair (M-TEER) of mitral regurgitation (MR) has emerged as an interventional therapeutic approach in selected patients and increased surgical risk [1–4]. The randomized controlled COAPT trial demonstrated beneficial effects of M-TEER on mortality and heart failure (HF) hospitalization [5].

An iatrogenic atrial septal defect (iASD) persists in roughly 24–50% of patients after transseptal access for M-TEER [6, 7] and is associated with impaired outcomes [6, 8–10]. In the MITHRAS-trial (Closure of Iatrogenic Atrial Septal Defect Following Transcatheter Mitral Valve Repair, NCT03024268) patients with an iASD and significant left-to-right shunting 1-month post-M-TEER were randomized to interventional closure of the iASD or conservative therapy (CT) [11]. While the presence of an iASD was independently associated with impaired outcomes, interventional closure did not improve the clinical course of the patients 6–12 months post-M-TEER implying the presence of an iASD to be rather an epiphenomenon than the causative source of clinical deterioration [11, 12]. Interestingly, some patients randomized to CT showed a decrease in shunt flow across the iASD between 1 and 6 months post-M-TEER even without interventional closure. Until today it remains unclear, whether a reduction of iASD shunt flow translates into improved clinical outcomes and if a spontaneous closure at 6 months follow-up can be anticipated by imaging characteristics of the iASD.

We therefore investigated echocardiographic features of the iASD in the CT cohort, its natural history and predictors of spontaneous iASD closure between 1 and 6 months post-M-TEER. Additionally, we evaluated the association of outcomes (HF hospitalization) with spontaneous closure when compared to patients with residual iASD.

## Methods

### Trial design

Details regarding the MITHRAS-trial have been published previously [11, 12]. In brief: in an investigator-initiated, randomized, unblinded trial, patients with a relevant iASD and predominantly left-to-right-shunting 1 month after interventional M-TEER were randomized in a 1:1 fashion to interventional closure of the iASD or CT. All patients underwent transthoracic (TTE) and transesophageal echocardiography (TEE) 1 month post-M-TEER and patients randomized to CT additionally at 6 months. A six minute walk test was performed at the day of the TTE/TEE and a routine testing of creatine, estimated glomerular filtration rate and N-terminal prohormone of brain natriuretic peptide (NT-proBNP). The trial was performed in accordance with the principles of the Declaration of Helsinki, the protocol was approved by the local ethics committee and all patients gave written informed consent. The trial was previously registered at Clinicaltrials.gov (NCT 03024268) and was funded by the Leipzig Heart Institute (Leipzig, Germany) and Occlutech (Jena, Germany).

### Study population

Consecutive M-TEER patients underwent 1 month follow-up assessment including TTE and TEE as previously described [12]. Patients could be included and randomized in case of an echocardiographic relevant iASD (fraction of pulmonary perfusion [Qp]/ fraction of systemic perfusion [Qs] ≥ 1.3) and predominantly left-to-right shunting 1-month post M-TEER. Exclusion criteria were significant left-to-right shunting (> 30%) prior to M-TEER, right-to-left shunting, unsuccessful M-TEER with residual MR grade > II.° and anatomic considerations precluding interventional iASD closure. The trial ended regularly after inclusion of the planned number of patients.

The here described subgroup analysis was performed in the CT arm of the MITHRAS trial, including patients with sufficient TEE 6 months post-M-TEER.

### Echocardiographic protocol

TTE and TEE (Vivid E9/E95, General Electric Healthcare, Chalfont St. Giles, Great Britain) were performed by experienced cardiologists. Chamber sizes, origin and degree of MR post-M-TEER and the severity of tricuspid regurgitation were classified according to the recommendations of the American Society of Echocardiography [13–15]. Intracardiac shunting through the iASD was quantified by the ratio of Qp:Qs [16]. Therefore, Qp was calculated by the perfusion through the shunt plus Qs. In TEE, iASD shunt flow was measured by multiplying the area of the iASD by the velocity time integral (VTI) through the iASD on continuous wave Doppler [17]. The area of the iASD was determined in 3D TEE. Qs was measured as diameter of the left ventricular (LV) outflow tract multiplied by the VTI of the pulsed-wave Doppler [16]. Due care was taken to acquire only the flow through the iASD. As an estimate of right ventricular (RV) systolic pressure, the RV to right atrium pressure gradient was calculated from the tricuspid regurgitation jet (without addition of right atrial pressure) [18].

The main aspects of the iASD were measured during the widest extension in 2 major dimensions including a major lengthwise (length) and a major oblique (width) dimension. An eccentricity index was calculated by dividing the lengthwise dimension by the oblique dimension (Graphical Abstract).

### Statistical analysis

Data for continuous variables are presented as mean ± standard deviation, if normally distributed, or as median and interquartile range (IQR) if non-normally distributed. Distribution was tested using Shapiro–Wilk tests. ANOVAs or Mann Whitney U tests were used to compare continuous variables. Categorical variables were compared with Chi^2^-tests and, if an assumption of ordinal scales was appropriate, p for trend is given. Kaplan–Meier analyses were used to compare the survival times in different subgroups; log-rank tests were used to test for differences.

Binary logistic regression analysis for the prediction of iASD closure was done including continuous and categorical variables. A receiver-operating characteristic analysis was performed to calculate thresholds and the area under the curve (AUC).

All time-to-event endpoints were defined with time of randomization as time zero. A *p*-value of less than 0.05 was considered statistically significant. Statistical analyses were performed using SPSS 28.0 (IBM, Armonk, USA).

## Results

### Baseline characteristics

A total of 40 patients, who underwent M-TEER at the Heart Center Leipzig at Leipzig University, Germany between January 2016 and October 2019, were randomly assigned to CT, of whom 36 received 1- and 6-months TEE (1 patient died prior to 6 months follow-up, 3 patients were hospitalized outside of our institution due to cardiac decompensation) composing the cohort for this subgroup analysis.

The median age was 77 (IQR 65–81) years, and the cohort included 36% female patients. EuroScore II was 5.5% (IQR 2.6–8.3), functional MR was present in 64% and a reduction of MR to grade 0-I was achieved in 72% and to grade II in 28% of the patients. One month after M-TEER, 33% of patients were in NYHA class I or II. Between 1 and 6 months follow-up, a total of 6 (17%) patients experienced spontaneous iASD closure as compared to 30 (83%) patients with residual iASD after M-TEER.

As shown in Table 1, patients in the spontaneous closure group had a higher New York Heart Association (NYHA) class 1 month after M-TEER (NYHA III + n = 6 [100%] vs n = 18 [60%], p = 0.04) and higher rates of single chamber implantable cardioverter defibrillators (ICDs).

**Table 1:** Baseline Characteristics 1 month after M-TEER

	Spontaneous iASD closure	iASD persistence	p-value
	n = 6	n = 30	
Age, *y*	78 (71–80)	72 (60–91)	0.70
Female sex, *no. (%)*	2 (33)	11 (37)	1.0
Diabetes, *no. (%)*	2 (33)	11 (37)	1.0
Hypertension, *no. (%)*	5 (83)	27 (90)	0.54
Hypercholesterolemia, *no. (%)*	3 (50)	25 (83)	0.11
Previous myocardial infarction, *no. (%)*	1 (17)	7 (23)	1.0
Previous coronary-artery bypass grafting, no. *(%)*	1 (17)	4 (13)	1.0
Previous stroke or transient ischemic attack, *no. (%)*	1 (17)	2 (7)	0.43
Peripheral vascular disease, *no. (%)*	0 (0)	2 (7)	1.0
Chronic obstructive pulmonary disease, *no. (%)*	0 (0)	6 (20)	0.56
History of atrial fibrillation of flutter, *no. (%)*	3 (50)	22 (73)	0.34
Body-mass index, *kg/m*^*2*^	26 (24–31)	24 (22–25)	0.08
Creatinine clearance median, ml/min/m^2^	40 (29–50)	58 (42–52)	0.29
EuroSCORE II,	5.5 (2.7–8.1)	5.6 (2.2–10.8)	0.93
NYHA class, *no. (%)*			**0.038**
I	0 (0)	0 (0)	
II	0 (0)	12 (40)	
III	5 (93)	17 (57)	
IV	1 (7)	1 (3)	
Pacemaker, *no. (%)*	0 (0)	1 (3)	1.0
Single-chamber-ICD device, no. *(%)*	4 (67)	4 (13)	**0.014**
CRT-D device, no. *(%)*	1 (17)	3 (10)	0.53
NT-proBNP, *ng/l*	3407 (1770–7638)	4108 (2395–5701)	0.86
Peripheral edema, *no. (%)*	4 (67)	17 (57)	1.0
Functional mitral regurgitation, %	3 (50)	20 (67)	0.65
Procedure time, min	40 (21–75)	70 (40–97)	0.064

*iAS*D iatrogenic atrial septal defect, *NYHA* new york heart association, *RV* right ventricular, *NT*-proBNP N-terminal prohormone of brain natriuretic peptide, *ICD* implantable cardioverter defibrillator, *CRT* cardiac resynchronization therapy, *LV* left ventricular

Indicated p-values are derived from Mann-U-Fisher exact test across between the groups

The MitraClip system (Abbott, Illinois, USA) was used in all patients of the spontaneous closure and in 28 (95%) patients of the residual shunt group. In two patients M-TEER was performed with the PASCAL system (Edwards Live Science, Irvine, USA). There was a trend towards shorter clip procedure times, measured in minutes starting with transseptal puncture to the retrieval of the delivery sheath to the right atrium, in the spontaneous closure patients (Table 1).

M-TEER resulted in a successful reduction of MR to grade ≤ 2 in all of the patients. The median MR reduction was 2 grades (p = 0.81 across groups). Overall, 66% of the patients in the spontaneous closure group and 57% of the patients in the residual iASD group received 2 clips. Only patients in the residual iASD group were treated with a 3-clip-strategy (n = 4 [13%], p = 0.64, (Supplemental Table 1).

### Baseline echocardiographic characteristics of iASD

**Fig. 1:**
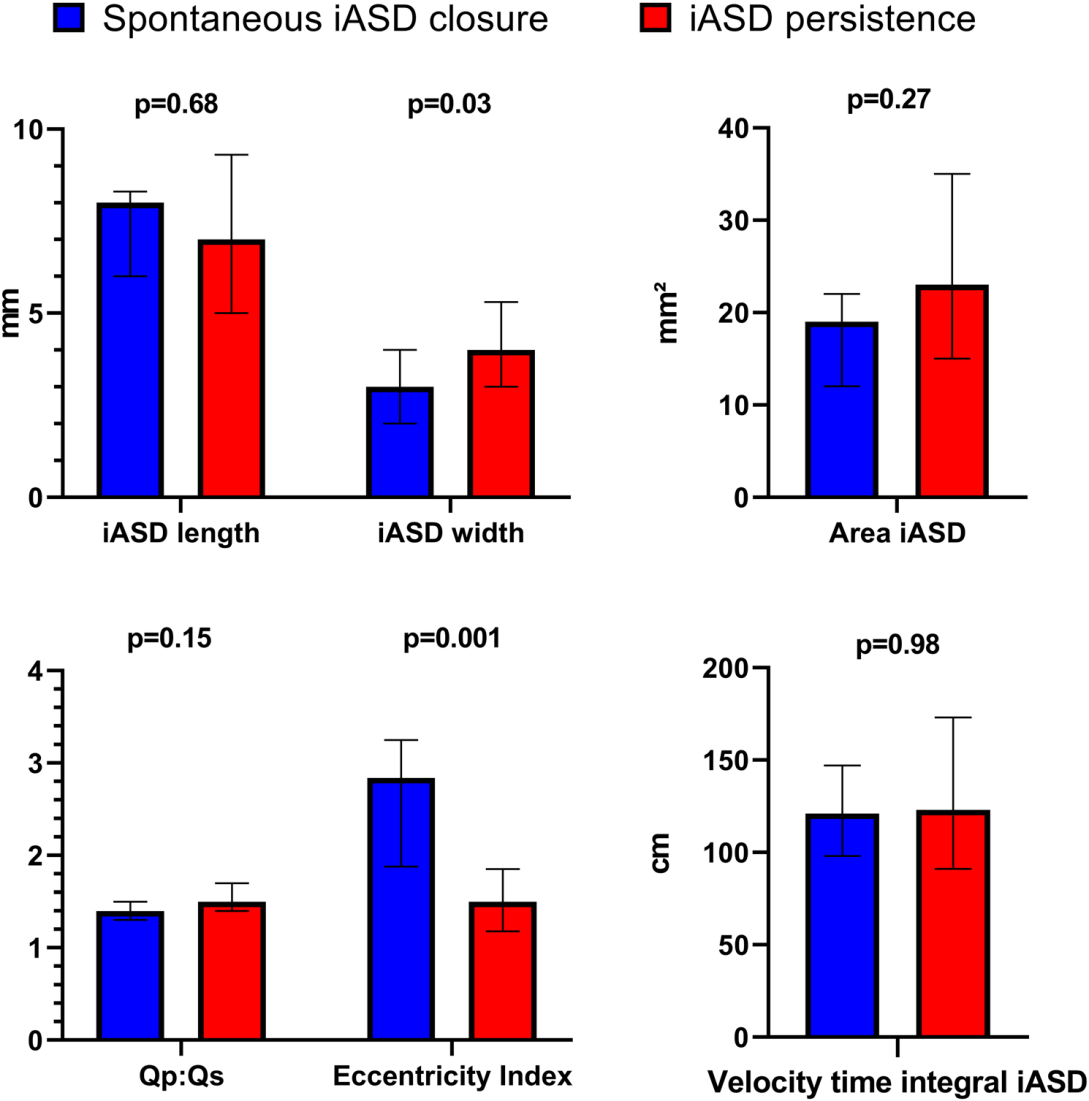
Comparison of echocardiographic characteristics of the iASD 1 month after M-TEER between the spontaneous iASD closure and iASD persistence group. Differences in transesophageal echocardiographic iASD characterization 1 month after M-TEER between patients with spontaneous iASD closure vs. iASD persistence at 6 months post-M-TEER p-values derived from ANOVAs or Mann Whitney U tests. Abbreviations: iASD: iatrogenic atrial septal defect, Qp: pulmonary perfusion, Qs: systemic perfusion, M-TEER: mitral transcatheter edge-to-edge repair

The area of the iASD did not differ between patients with a spontaneous closure as compared to those with persistence (19 [IQR 12-22] vs 23 [IQR 15-36] mm^2^, p = 0.29). However, while no differences in the length of the iASD was observed (8.0 [IQR 6.0–8.3] vs 7.0 [IQR 5.0–9.3] mm, p = 0.68), iASD-width was smaller in patients with a spontaneous closure (3.0 [IQR 2.0–4.0] vs 4.0 [IQR 3.0–5.3] mm, p = 0.03), resulting in a significantly higher eccentricity index in the closure-group (2.8 [IQR 1.9-3.3] vs 1.5 [IQR 1.2-1.9] mm, p = 0.01). Blood flow through the shunt was similar in both groups (23 [IQR 14-30] vs 26 [IQR 18-41] ml, p = 0.42), as well as Qp:Qs (1.4 [IQR 1.3-1.5] vs 1.5 [IQR 1.4-1.7], p = 0.15) (Table 2 and Fig. 1).

**Table 2:** Echocardiographic characteristics 1 month and 6 month after M-TEER

	1 month post M-TEER			6 month post M-TEER		p-value
	Spontaneous iASD closure	iASD persistence	p-value	Spontaneous iASD closure	iASD persistence	
	n = 6	n = 30		n = 6	n = 30	
Qp:Qs	1.4 (1.3–1.5)	1.5 (1.4–1.7)*	0.154	X	1.4 (1.2–1.5)*	
Eccentricity Index	2.84 (1.88 – 3.25)	1.50 (1.18 – 1.85)	**0.001**	X	1.37 (1.20 – 1.67)	
LVEF, %	42 (18—64)	33 (24—53)	0.81	41 (21—60)	34 (27—54)	0.88
LVEDV, *ml*	192 (60 – 313)	171 (126 – 227)	0.76	149 (73 – 300)	164 (120 – 214)	0.64
TAPSE, mm	12 (9–18)	16 (12–20)	0.19	16 (12–19)	15 (13–19)	0.91
RV Base Diameter, mm	38 (33–40)*	39 (35–41)	0.43	33 (30 – 35)*	37 (34 – 45)	0.007
ePAP, mmHg	33 (30—41)	41 (36—48)	0.086	34 (29—41)	39 (30—46)	0.70
LA volume, ml	61 (57—73)	69 (53—82)	0.54	59 (53—68)	71 (56—79)	0.19
RA volume, ml	58 (30—84)	53 (41—67)	0.81	38 (27—71)	46 (32—66)	0.63
Pmax iASD, mmHg	25 (19—29)	21 (14—30)	0.61	X	18 (14—28)	
Pmean iASD, mmHg	13 (7—18)	12 (7—17)	0.70	X	10 (7—13)	
Flow through iASD, ml per heartcycle	22.8 (14.1 – 30.4)	25.5 (18.0 – 41.3)	0.42	X	17.2 (9.1 – 35.5)	
VTI iASD, mm	121 (98—147)	123 (91—173)	0.98	X	115 (95—145)	
Area iASD, mm^2^	19 (12—22)	23 (15—35)*	0.27	X	16 (7—32)*	
Length iASD, mm	8.0 (6.0–8.3)	7.0 (5.0 – 9.3)	0.68	X	6.0 (3.8 – 8.0)	
Width iASD, mm	3.0 (2.0–4.0)	4.0 (3.0 – 5.3)	**0.03**	X	4.0 (2.8 – 5.3)	
MV mean gradient, mmHg	4.0 (3.2–5.0)	4.0 (3.0—4.0)	0.09	4.0 (3.2 – 5.0)	3.5 (3.0—4.0)	0.05
MR grade, n (%)			0.69			0.78
0–I	5 (84)	21 (70)		4 (66)	17 (57)	
II	1 (16)	9 (30)		2 (33)	13 (43)	
III	0	0		0	0	
TR grade, n (%)			0.191			0.64
0–I	3 (50)	15 (50)		4 (66)	18 (60)	
II	2 (33)	12 (40)		1 (17)	7 (23)	
≥ III	1 (17)	3 (10)		1 (17)	5 (17)	

*iASD* iatrogenic atrial septal defect, *RV* right ventricular *LV* left ventricular, *EF* ejection fraction, *EDV* enddiastolic volume, *Qp* fraction of pulmonary perfusion, *Qs* fraction of systemic perfusion, *TAPSE* tricuspid annular plane systolic excursion, *MV* mitral valve, *ePAP* estimated systolic pulmonary artery pressure, *MR* mitral regurgitation, *TR* tricuspid regurgitation, *VTI* Velocity time integral

Indicated p-values are derived from Mann-U-Fisher exact test across between the groups, *marked parameters have p-values < 0.05 within group comparison

Bold parameters have p-values < 0.05 between group comparison

### Echocardiographic changes of iASD

In the group without spontaneous closure, a significant reduction of the iASD-area was observed between 1- and 6-month post-M-TEER (23 [IQR 15-36] vs 16 [IQR 8-33] mm^2^, p = 0.02), driven by a reduction of the iASD length and to a lesser extent of the width. No change in the VTI was observed. Accordingly, the observed reduction of Qp:Qs was mainly driven by a reduction of the iASD size (Table 3).

**Table 3:** Echocardiographic characteristics of the iASD 1 month and 6 month after M-TEER in the iASD persistence group

	1 month post M-TEER	6 month post M-TEER	p-value
	n = 30	n = 30	
Qp:Qs	1.5 (1.4–1.7)	1.4 (1.2–1.5)	**0.016**
Eccentricity Index	1.50 (1.18 – 1.85)	1.37 (1.20 – 1.67)	0.27
LVEF, %	33 (24–53)	34 (27–54)	0.22
LVEDV, *ml*	171 (126 – 227)	164 (120 – 214)	0.81
TAPSE, mm	16 (12–20)	15 (13–19)	0.46
RV Base Diameter, mm	39 (35–41)	37 (34–45)	0.30
ePAP, mmHg	41 (36–48)	39 (30–46)	0.35
LA volume, ml	69 (53–82)	71 (56–79)	0.31
RA volume, ml	53 (41–67)	46 (32–66)	0.32
Pmax iASD, mmHg	21 (14–30)	18 (14–28)	0.19
Pmean iASD, mmHg	12 (7–17)	10 (7–13)	0.50
Flow through iASD, ml	2552 (1797–4135)	1720 (901–3548)	0.08
VTI iASD, mm	123 (91–173)	115 (95–145)	0.83
Area iASD, mm^2^	23 (15–35)	16 (7–32)	**0.02**
Length iASD, mm	7.0 (5.0–9.3)	6.0 (3.8–8.0)	**0.003**
Width iASD, mm	4.0 (3.0–5.3)	4.0 (2.8–5.3)	**0.05**
MV mean gradient, mmHg	4.0 (3.0–4.0)	3.5 (3.0–4.0)	0.21
MR grade, n (%)			0.42
0–I	21 (70)	17 (57)	
II	9 (30)	13 (43)	
≥ III	0	0	
TR grade, n (%)			1.0
0–I	15 (50)	18 (60)	
II	12 (40)	7 (23)	
≥ III	3 (10)	5 (17)	

*iASD* iatrogenic atrial septal defect, *RV* right ventricular *LV* left ventricular, *EF* ejection fraction, *EDV* enddiastolic volume, *Qp* fraction of pulmonary perfusion, *Qs* fraction of systemic perfusion, *TAPSE* tricuspid annular plane systolic excursion, *MV* mitral valve, *ePAP* estimated systolic pulmonary artery pressure, *MR* mitral regurgitation, *TR* tricuspid regurgitation, *VTI* Velocity time integral

Indicated p-values are derived from Mann-U-Fisher exact test

Bold parameters have p-values <0.05 for the within group comparison

### Predictors of spontaneous iASD closure

Univariable and stepwise binary logistic regression analyses revealed eccentricity index as strong predictor of spontaneous iASD closure (adjusted Odds ratio 3.78, 95% confidence interval 1.25–11.33, p = 0.018) (Table 4).

**Table 4:** Binary logistic regression predicting spontaneous iASD closure at 6 months after M-TEER

	Univariate		Stepwise Multivariable				
	Chi Square	p-value	R^2^	β	Odds Ratio	Confidence interval	p-value
Eccentricity Index 1 month, per 1 unit	6.592	**0.01**	0.282	1.33	3.78	1.261 – 11.33	**0.018**
Width 1 month, per 1 mm	5.498	**0.02**	0.238	-0.863	0.442	0.169–1.052	0.064
MV mean 1 month, per 1 mmHg	4.197	**0.04**	0.185	-0.906	0.404	0.149–1.093	0.074
Procedure Time, per 1 min	3.69	**0.06**	0.164	-0.029	0.971	0.938–1.006	0.104
Qp:Qs 1 month, per 1 unit	2.49	0.11					
iASD Area 1 month, per 1 mm^2^	2.179	0.14					
TAPSE 1 month, per 1 mm	2.07	0.15					
ePAP 1 month, per 1 mmHg	1.88	0.17					
TR 1 month, per 1 grade	5.5	0.24					
Flow ASD 1 month, per 1 unit	1.259	0.26					
RV Diameter, per 1 mm	0.648	0.42					
LV EF 1 month, per 1%	0.45	0.50					
LA Vol 1 month, per 1 mm^3^	0.444	0.51					
MR 1 month, per 1 grade	0.442	0.51					
Number of Clips, per 1 unit	0.372	0.54					
iASD VTI 1 month, per 1 mm	0.098	0.76					
Pmean iASD 1 month, per 1 mmHg	0.06	0.80					
RA Volume 1 month, per 1 mm^3^	0.038	0.85					
Age, per 1 year	0.027	0.87					
Sex (Reference female)	0.024	0.88					
Euroscore II, per 1 unit	0.006	0.94					
Pmax ASD 1 month, per 1 mmHg	0.003	0.96					
Length iASD 1 month, per 1 mm	0.003	0.96					

*iASD* iatrogenic atrial septal defect, *RV* right ventricular *LV* left ventricular, *EF* ejection fraction, *Qp* fraction of pulmonary perfusion, *Qs* fraction of systemic perfusion, *TAPSE* tricuspid annular plane systolic excursion, *MV* mitral valve, *ePAP* estimated systolic pulmonary artery pressure, *MR* mitral regurgitation, *TR* tricuspid regurgitation, *VTI* Velocity time integral

Indicated p-values are derived f. Nagelkerkers estimated R^2^ is given

Bold parameters have p-values < 0.1 in the univariate, respectively a p-value < 0.05 in the stepwise multivariable binary logistic regression analysis

**Fig. 2:**
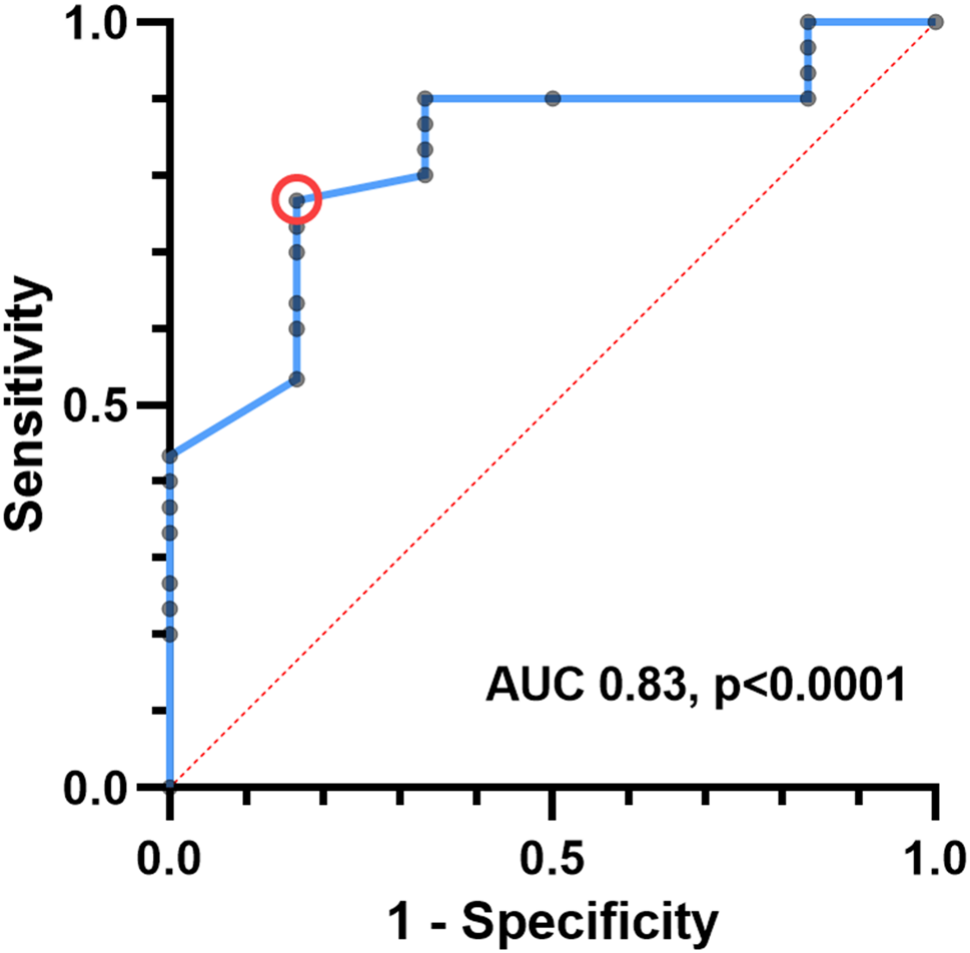
Receiver-operator curves on eccentricity index for iASD persistence. An eccentricity index of < 1.9 (red circle) provided a sensitivity of 77% at a specificity of 83% for iASD persistence, ROC with an AUC 0.83 and a p < 0.0001. Abbreviations: AUC: area under the curve, iASD: iatrogenic atrial septal defect, ROC: receiver operated curves

Receiver-operating characteristic analysis revealed good prediction of the eccentricity index for iASD persistence (AUC 0.83, p < 0.0001) and an eccentricity index of < 1.9 provided a sensitivity of 77% at a specificity of 83% for iASD persistence (Fig. 2).

### Clinical and echocardiographic outcomes

Follow-up regarding the combined endpoint was available for all patients at 6 months (median follow-up time 183 days [IQR 180–192]) after M-TEER.

#### Echocardiographic results in comparison to 1 month post M-TEER

In the spontaneous iASD closure group, the RV basal diameter declined in comparison to the baseline echocardiography (38 [IQR 33-40] vs 33 [IQR 30-35] mm, p = 0.043) and a numerical rise in TAPSE could be observed (12 [IQR 8.5-17.5] vs 16 [IQR 11.75-18.75] mm, p = 0.058). Besides this, no significant changes regarding other chamber diameters, LV function or pulmonary artery pressures (PAPs) were observed. Chamber diameters, LV function and PAPs remained unchanged in comparison to baseline evaluation.

Reduction of the MR and tricuspid regurgitation maintained at 6-month follow-up without any significant differences compared to 1-month post-M-TEER (Table 2).

#### Clinical outcomes

Severe HF symptoms (NYHA class III/IV) at 6-months did not differ between groups. (Spontaneous closure group vs. residual shunt group NYHA III + n = 2 [33%] vs n = 9 [30%], p = 0.41).

All patients completed a six-minute walk test 1-month and 6-months post-M-TEER. No significant changes in the walking distance could be observed in both groups [iASD spontaneous closure 1-month post-M-TEER vs 6-month post-M-TEER 333 (IQR 276-452) vs 397 (IQR 265-505) meter) and residual shunt group 1-month post-M-TEER vs 6-month post-M-TEER 287 (IQR 196-406) vs 284 (IQR 203-375) meter, with a Δ of 52 (IQR 29–75) vs -12 (IQR 36-40) meter] without reaching statistical significance between and within groups.

Glomerular filtration rate increased significantly in both cohorts at 6-month post M-TEER compared to baseline measurements (spontaneous iASD closure group 48 (IQR 42-52) vs 62 (IQR 47-88) ml/min/m^2^, p = 0.042, iASD persistence group 40 (IQR 29-0) vs 42 (IQR 35-63) ml/min/m, p = 0.006) but did not differ between groups at baseline (p = 0.29) and 1-month follow-up (p = 0.12), respectively.

NT-proBNP levels were available for 33 patients (6 spontaneous closure group and 27 in the residual shunt group) 1-month and 6-months post-M-TEER. In trend, NT-proBNP decreased in both groups between timepoints [iASD spontaneous closure 1-month post-M-TEER vs 6 months post-M-TEER 3407 (IQR 1770-7639) vs 2679 (IQR 1616-7772) ng/ml) and residual shunt group 1-month post-M-TEER vs 6-months post-M-TEER 4108 (IQR 2395-5701) vs 3414 (IQR 1418-5362) ng/ml, with a Δ of − 326 (IQR 1996-2418) vs − 350 (IQR 987-280) ng/ml) without reaching statistical significance between or within groups.

**Fig. 3:**
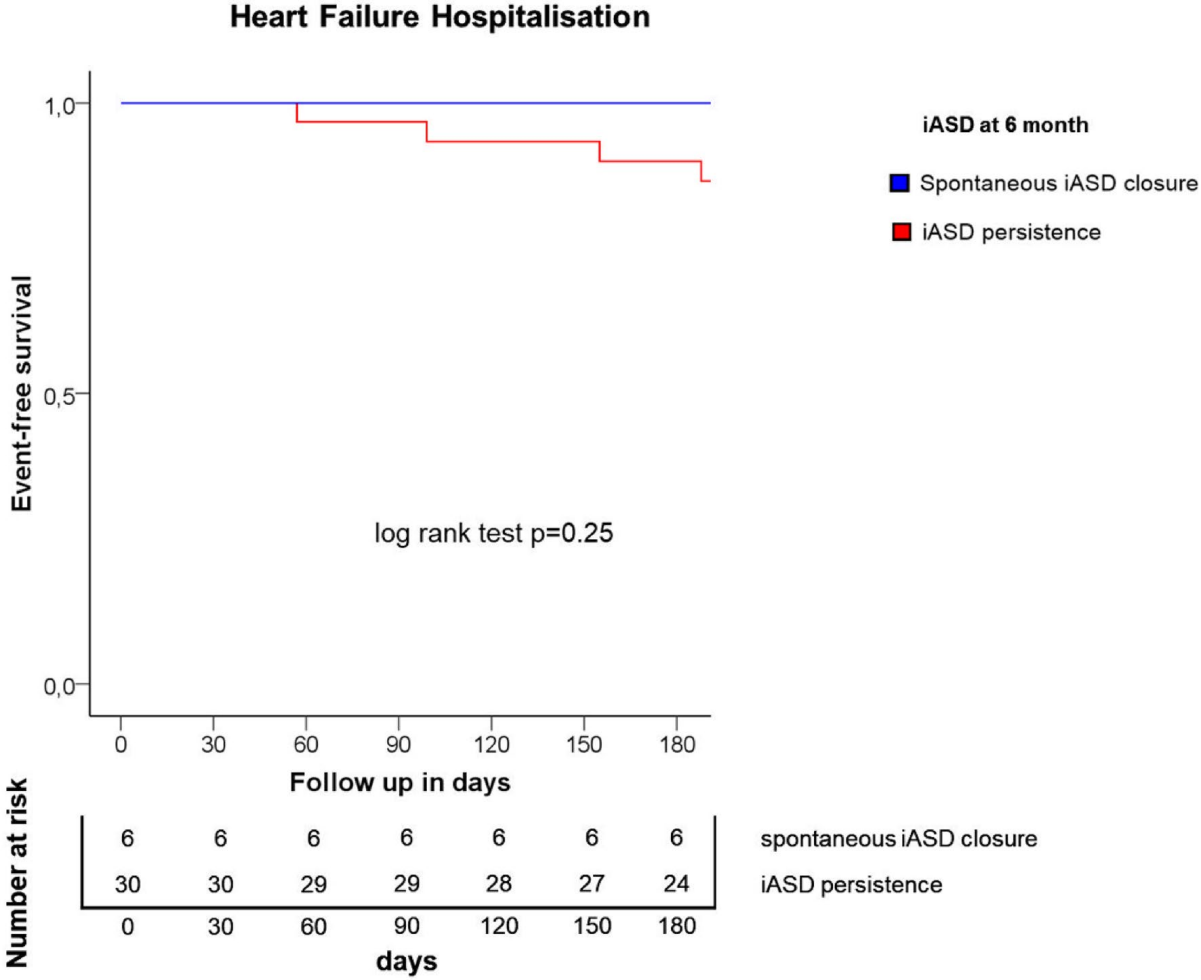
Kaplan–Meier survival curves for endpoint of heart failure hospitalization at 6-month follow-up post M-TEER. Kaplan–Meier estimated event rates in the spontaneous iASD closure and the residual shunt group for the time to the endpoint of heart failure hospitalization. Abbreviations: iASD: iatrogenic atrial septal defect

The endpoint of HF hospitalization at 6-months follow up showed no significant differences between the groups (spontaneous iASD closure vs residual shunt group 0 [0%] vs 6 [20%], p = 0.25) (Fig. 3).

## Discussion

To date, this is the first study to describe the fate of significant iASDs post-M-TEER with serial 3D TEE to elucidate the geometrical shape of the iASD and its impact on the spontaneous closure probability.

In this subgroup analysis of the MITHRAS trial, the strongest predictor for spontaneous closure was eccentricity of the iASD 1-month after M-TEER. Whereas an eccentricity index of < 1.9 predicts a persistence of the iASD 6 months post-M-TEER with great accuracy. Notably, patients with a spontaneous closure of the iASD had a numerical lower risk for HF hospitalization at 6-month follow-up.

Currently, there is no guideline-based recommendation on whether or when to close a postinterventional iASD following M-TEER. In the MITHRAS trial the closure of an iASD 1-month after M-TEER did not have an effect on clinical (death / HF hospitalization) or functional outcomes at 5 months post closure. However, patients with a persistent iASD experienced worse outcomes 12-months post-M-TEER as compared to patients without an iASD post-M-TEER [11, 12]. Of note, the effects of interventional iASD closure on the event rates may have been diminished by spontaneous closure.

### Persistence of iASD after M-TEER

iASD has been described to persist in 27% to 35% of patients at 6 to 12 months when assessed by TTE and in 42% to 50% of cases when using TEE as a more sensitive method [6, 19–21].

The prevalence of iASD might be underestimated when using TTE imaging. Importantly the geometric characterization is only sufficiently possible with 3D TEE [17]. Therefore, our results are not completely comparable to the aforementioned cohorts. Importantly, we included patients with predominant left-to-right shunting, while other studies described the overall persistence including right-to-left-shunting.

### Possible mechanisms for spontaneous closure

Two studies linked the presence of an iASD to higher left atrial pressures due to higher MR or to the presence of tricuspid regurgitation [19, 21]. One study suggested that, due to tricuspid regurgitation, the septal mobility is increased and therefore the healing process may be disturbed [7]. In our cohort no differences between the groups in MR or tricuspid regurgitation, as well as echocardiographic estimated PAP could be observed 1 month or 6 months after M-TEER.

Procedure time, device time and catheter manipulation at the atrial septum were presented as the main factors for an iASD in two studies. [6, 20] Procedure time of 71 ± 39 min was not associated with the size of the iASD and was more comparable to that of the non iASD group (69 ± 46 min) than to the iASD group (83 ± 40 min) in the Schueler cohort [6]. However, the procedure time was numerical lower in the spontaneous iASD closure group in comparison to the iASD persistence group (40 (IQR 21-75) minutes vs. 70 (IQR 40-97) minutes, p-value 0.064) in our cohort. Extended exposure of the atrial septum to sheer and torque pressure, due to longer catheter manipulation time, may lead to rounder defects which were associated with persistence of the iASD.

In line with published results, no correlation between shunt volume, iASD diameter or size and persistence could be observed in the current cohort [6, 17, 20, 21]. Two spontaneous closures of very eccentric iASDs within 30 days after M-TEER could be documented by Saitoh et al.[17] These observations can possibly be explained by a ripped shape of the iASD, as our findings unraveled a strong correlation between the eccentricity of the iASD and the closure probability. Leading to the assumption that eccentric torn defects have a longer coaptation line of the iASD defect border, thus facilitating healing in comparison to rounder pierced defects. Supporting this, the reduced length but stable width of the iASD at 6-months follow-up underlines the theory that healing starts at the vertices of the major axis.

### Functional outcomes and clinical event rates

#### Functional and clinical outcomes

While functional and clinical outcomes previously reported were worse in patients with an iASD, the results are prone to bias due to unrandomized fashion of the data and the inclusion of patients with right-to-left shunting [6]. Conclusive data based on TEE-based examinations are lacking and we could not detect clinically meaningful baseline differences in patients with a spontaneous iASD closure as compared to those with a persistence. However, given the relatively small patient cohort the results must be interpreted with caution. We found a trend towards improved RV function in patients with spontaneous iASD closure. While acute occlusion of an iASD does not improve markers of load-independent RV function, chronic volume relieve of the RV might be beneficial [22]. However, given the small number of patients, this result must be interpreted with caution.

#### Comparison to interatrial shunt devices

Interatrial shunt devices were a large spark of hope for a high number of patients with HF. Promising early results of the REDUCe Elevated Left Atrial Pressure in Patients with Heart Failure (REDUCE LAP-HF), with regards to symptoms and hemodynamics have been reported and have led to the initiation and completion of the REDUCE LAP-HF II trial, which failed to show statistical significance for its primary endpoint [9, 10]. It is tempting to compare the data of the REDUCE LAP-HF II trial to our data but there are several differences that are important to keep in mind before doing so.

First of all, the theoretical concept of an iASD device is reducing left atrial pressure by decongesting the left atrium as flow is produced from the left atrium to the right atrium. This in line, increases the right atrial pressure and might cause RV deterioration. An iASD diameter of 8 mm was identified as the optimal trade-off in theoretical models [23]. In line with this, patients with RV dysfunction were excluded from the study. Further, the study showed that there was a subgroup of HF with preserved ejection fraction patients where pulmonary vascular resistance was normal at exercise that seemed to benefit from an iASD making a thorough assessment of RV function crucial when investigating whether a patient might benefit from an iASD [10]. Patients in our cohort had a high frequency of evident RV dysfunction even in the absence of more profound hemodynamic assessment making a favorable effect of an iASD unlikely. We previously reported on beneficial hemodynamic effects of iASD closure in patients with HF and mainly reduced ejection fraction, a group excluded from REDUCE LAP-HF II, which is again an important point to distinguish both concepts [22]. Lastly while the Qp:Qs in the iASD persistence group was 1.2 to 1.5, the Qp:Qs ratio in REDUCE-LAP II was 1.2 to 1.3, the iASD area in our cohort was 15 to 35 mm^2^ and 50 mm^2^ (assuming a device diameter of 8 mm) in the REDUCE LAP II trial. This lower flow, despite significantly higher areas, indicates a significantly smaller driving pressure gradient making comparison of both groups unreliable. Lastly, a new device which investigates an iASD creation in both HF patients with preserved or reduced ejection fraction with a smaller diameter of 5 mm, has shown interesting data and will likely be more comparable to our cohort, but recruitment is still ongoing [24].

### Limitations

The sample size, especially those of the spontaneous closure cohort, is rather small and the conclusions drawn should be seen as hypothesis generating, as statistical power is lacking to exclude effects not observed. The shunt fraction of 30% has been proposed as a cut-off to balance the risk of RV distension due to volume overload to the benefit of alleviating left atrial pressure by an intentionally created ASD in patients with HF and was therefore chosen as inclusion criterion in the randomized MITHRAS trial [23]. Given the small sample size we cannot exclude that fate of the iASD in patients with specific MR etiologies (functional or degenerative), higher degrees of concomitant TR or RV dysfunction may take another progression.

## Conclusion

In this subgroup analysis of the MITHRAS trial, the strongest predictor for spontaneous closure was the eccentricity of the iASD 1 month after M-TEER and an eccentricity index < 1.9 predicts with great accuracy a persistence of the iASD 6 months post-M-TEER.

## Impact on daily practice

Patients with a significant (Qp:Qs > 1.3) but eccentric iASD (eccentricity index ≥ 1.9) 1 month after M-TEER have a higher closure probability within 6 months in comparison to patients with rounder defects. The geometric characterization of the iASD maybe a viable strategy to improve the timing of treatment (interventional closure vs. watchful waiting).

## Supplementary Information

Below is the link to the electronic supplementary material.

Supplementary file1 (DOCX 12 kb)

## Abbreviations

AUC: Area under the curve
CT: Conservative therapy
HF: Heart failure
iASD: Iatrogenic atrial septal defect
ICD: Implantable cardioverter defibrillators
IQR: Interquartile range
LV: Left ventricle / left ventricular
MR: Mitral regurgitation
NT-proBNP: N-terminal prohormone of brain natriuretic peptide
NYHA: New York Heart Association
PAP: Pulmonary artery pressure
Qp: Pulmonary perfusion
Qs: Systemic perfusion
RV: Right ventricle / right ventricular
M-TEER: Transcatheter edge-to-edge repair
TTE / TEE: Transthoracic / transesophageal echocardiography
VTI: Velocity time interval
