# Supplementary material for: Fate of iatrogenic atrial septal defects following mitral transcatheter edge-to-edge repair – a subanalysis of the MITHRAS trial
Source: Int J Cardiovasc Imaging. 2022 Nov 13;39(3):519–30. doi: 10.1007/s10554-022-02750-5 (PMC9947030; doi:10.1007/s10554-022-02750-5)
Supplement: Supplementary file 1 — Supplementary file1 (DOCX 12 kb) [file 10554_2022_2750_MOESM1_ESM.docx]

**Supplemental Table 1**

Number of Clips

|  | **Spontaneous iASD closure** | | **iASD persistence** | p-value |
| --- | --- | --- | --- | --- |
|  | n=6 | | n=30 |  |
|  | |  |  | 0.64 |
| 1*, no (%)* | | 2 (33) | 9 (30) |  |
| 2, *no (%)* | | 4 (66) | 17 (57) |  |
| 3, *no (%)* | | 0 (0) | 4 (13) |  |
